# What do District Health Planners in Tanzania think about improving priority setting using 'Accountability for Reasonableness'?

**DOI:** 10.1186/1472-6963-7-180

**Published:** 2007-11-12

**Authors:** Simon Mshana, Haji Shemilu, Benedict Ndawi, Roman Momburi, Oystein Evjen Olsen, Jens Byskov, Douglas K Martin

**Affiliations:** 1 Primary Health Care Institute, Iringa, Tanzania; 2 DBL- Institute for Health Research and Development, Charlottenlund, Denmark; 3 Department of Health Policy, Management and Evaluation and the Joint Centre for Bioethics, University of Toronto, Toronto, Canada; 4 deceased

## Abstract

**Background:**

Priority setting in every health system is complex and difficult. In less wealthy countries the dominant approach to priority setting has been Burden of Disease (BOD) and cost-effectiveness analysis (CEA), which is helpful, but insufficient because it focuses on a narrow range of values – need and efficiency – and not the full range of relevant values, including legitimacy and fairness. 'Accountability for reasonableness' is a conceptual framework for legitimate and fair priority setting and is empirically based and ethically justified. It connects priority setting to broader, more fundamental, democratic deliberative processes that have an impact on social justice and equity. Can 'accountability for reasonableness' be helpful for improving priority setting in less wealthy countries?

**Methods:**

In 2005, Tanzanian scholars from the Primary Health Care Institute (PHCI) conducted 6 capacity building workshops with senior health staff, district planners and managers, and representatives of the Tanzanian Ministry of Health to discussion improving priority setting in Tanzania using 'accountability for reasonableness'. The purpose of this paper is to describe this initiative and the participants' views about the approach.

**Results:**

The approach to improving priority setting using 'accountability for reasonableness' was viewed by district decision makers with enthusiastic favour because it was the first framework that directly addressed their priority setting concerns. High level Ministry of Health participants were also very supportive of the approach.

**Conclusion:**

Both Tanzanian district and governmental health planners viewed the 'accountability for reasonableness' approach with enthusiastic favour because it was the first framework that directly addressed their concerns.

## Background

Priority setting in every health system is complex and difficult. In wealthy countries the dominant approach to priority setting has been Health Technology Assessment (HTA) – evidence based medicine and cost-effectiveness – which is helpful, but insufficient because HTA focuses on only a narrow range of values – benefit and efficiency – and not the full range of values that are relevant to priority setting, including legitimacy and fairness[1]. Decision makers and scholars have utilized 'accountability for reasonableness' as a framework to facilitate deliberation about these and other relevant values and identify opportunities to improve priority setting practices[2,3]

'Accountability for reasonableness' is a conceptual framework for legitimate and fair priority setting. It was developed in the context of U.S. Health Maintenance Organizations [4], and so is relevant to real-world priority setting; it is theoretically grounded in justice theories emphasizing democratic deliberation [5,6]. According to 'accountability for reasonableness', priority setting is fair to the degree it meets four conditions:

### Relevance

Rationales for priority setting decisions must rest on reasons (evidence and principles) that stakeholders can agree are relevant in the context. Only participation by the full range of stakeholders can ensure that the full range of relevant reasons are brought to the deliberations.

### Publicity

Priority setting decisions and their rationales must be publicly accessible. Publicity means that leaders must take action to 'push' the message out to all segments of the public. Thus, publicity goes beyond mere transparency.

### Revisions

There must be a mechanism for challenge, including the opportunity for revising decisions in light of considerations that stakeholders may raise. This provides a quality assurance mechanism to difficult, and occasionally controversial, decision making and demonstrates responsiveness on the part of leaders.

### Enforcement/Leadership

Leaders in each context are responsible for ensuring that the first three conditions are met in their context.

'Accountability for reasonableness' is the only approach to priority setting that is empirically based, ethically justified, and focused on process. It can be used as an analytic lens to facilitate social learning about priority setting and it connects priority setting to broader, more fundamental, democratic deliberative processes that have an impact on social justice and equity. It has been used in wealthy countries to evaluate and improve priority setting in healthcare – for example, regions [7], hospitals [8], clinical programs[9,10], and drug formularies [11].

In less wealthy countries the dominant approach to priority setting has been Burden of Disease (BOD) and cost-effectiveness analysis (CEA), which is also helpful, but insufficient because it focuses on a narrow range of values – need and efficiency – and not the full range of relevant values, including legitimacy and fairness. Moreover, Kapiriri *et al*. evaluated priority setting in Uganda and found that the BOD/CEA approach: i) did not include other values that were important to Ugandans (e.g. protecting the vulnerable); ii) was too opaque; iii) did not involve relevant people, and iv) was too technical, requiring expertise that was unavailable[12]. Just as it has in wealthy countries, 'accountability for reasonableness' may be useful for improving priority setting in less wealthy countries. However, there have not been attempts to improve priority setting in less wealthy countries utilizing 'accountability for reasonableness'.

Can 'accountability for reasonableness' be helpful for improving priority setting in less wealthy countries?

In 2005, Tanzanian scholars from the Primary Health Care Institute (PHCI) partnered with colleagues from DBL-Institute for Health Research and Development (DBL – Denmark) and the University of Toronto Joint Centre for Bioethics (JCB – Canada) in a capacity building initiative to engage health planners in the Southern Highlands Zone, in and around Iringa, Tanzania, in discussions about improving priority setting using 'accountability for reasonableness'. PHCI is a Zonal Training Centre with a mandate to provide training of health professionals and health system management support to the districts of Iringa and Ruvuma Regions in Tanzania. This initiative involved 6 workshops with different groups consisting of senior health staff, including planners and managers.

The purpose of this paper is to describe an initiative in Tanzania to improve priority setting using 'accountability for reasonableness' and the participants' views about the approach.

## Methods

### Phase I

The purpose of the first phase was to introduce and explore the acceptability of the key concepts. It consisted of 2 workshops – one with planners, senior health staff and a few other providers (e.g. representatives of faith-based and voluntary organizations) and users of health services in Iringa District and Iringa Municipality, held in Iringa (n = 20), and one with representatives of the Tanzanian Ministry of Health, faith-based, voluntary, and international development organizations, held in Dar es Salaam (n = 20). The workshops consisted of didactic seminars that explored key issues relevant to priority setting and 'accountability for reasonableness', and small and large group discussions in which participants could express views and ask questions. The output of the first phase was high acceptance of the approach in both workshops, a description of health planners' needs related to priority setting and 'accountability for reasonableness', and a draft needs assessment tool. The needs assessment tool was then pre-tested in a district not otherwise involved (Njombe District).

### Phase II

The purpose of the second phase was to implement the needs assessment tool and capture the responses of participants to the key concepts and the approach. It consisted of 4 workshops – two in Ruvuma Region (Mbinga and Songea districts) and two in Iringa Region (Mufindi and Ludewa districts). Each workshop involved 19 participants from the district (total of 76 participants). The participants included key members of the District Health Planning Team, Civil Society through voluntary agencies and church groups, patient representatives, and identified respected members of the community. The workshops consisted of a series of questions asking participants to describe their views in relation to each aspect of the 'accountability for reasonableness' approach to priority setting.

## Results

### The views of Tanzanian Health Planners

This section summarizes the views of workshop participants organized according to Positive Views and Concerned Views. Verbatim quotes are included for illustration.

### Positive views

The participants in all 6 workshops demonstrated a high degree of acceptance of the approach and expressed enthusiasm toward implementing it in their particular context. Specific positive comments were related to three specific issues.

### The approach enables wider participation

A respected community member commented, "It was an eye opener . . . we did not know that we could be involved in setting priorities for the district." The observation was also made by a bureaucrat at the district level, who said, "Normally in my district it is only the DMO (District Medical Officer) and a few persons who sit and make the plan." A pharmacist revealed that it was his first time to be invited in a health issue: "I really appreciate the AFR initiative as it will give room to other stakeholders to contribute to the district health plan.

In addition, it was revealed that private not-for-profit sectors are not involved in the district planning process, although their involvement is mentioned in the Comprehensive Council Health Plan guideline.

### The approach enables greater transparency

Several participants commented that involving more stakeholders creates greater transparency – by being involved, more people develop a greater understanding of the district plans. For example a representative from the patients group *People Living with HIV/AIDS *revealed that they just heard that Antiretroviral drugs are available at the regional hospital, but that there was no clear information on how those drugs can be accessed, and they would have known this had they been more involved in decision making.

### The approach enables the scrutiny and development of relevant criteria

Current planning guidelines include criteria for priority setting, but the criteria are insufficient, not explicit, and not appropriate to all areas. Thus, implementing AFR during priority setting will provide room for inclusion of other values based on geographical or cultural variations and other factors.

### Concerned views

Some participants expressed concerns about implementing this approach in their context, which could be organized into 3 categories.

### Concerns about the approach

Some felt that the approach is technical and complicated. Others thought that it may be perceived as just another tool. Others felt concern that, for the approach to be implemented at district levels, it has to be approved by higher level officials (Presidents Office Regional and Local Government and the Ministry of Health). Some were concerned that the approach would create tensions between planners and politicians. A high ranking nurse said, "How do we explain to community 'reasons' for not including their priority in the district plan while a leader or an influential person goes around giving districts promises?"

### Concerns about stakeholder involvement

According to participants, many potential stakeholders do not have the knowledge, skills or experience to effectively contribute to priority setting decisions. Some participants who had never participated in priority setting worried that they may be too intimidated to effectively participate. Even for planners, more time was believed to be needed for sensitization and capacity building. In addition, some were concerned that incorporating additional stakeholders would add costs that could not be justified in the current planning frame.

### Concerns about developing relevant reasons

Most felt that communication of reasons is complex and difficult. In some decision making contexts, the criteria to be used in setting priorities during development of Council Comprehensive Health Plan have already been set by higher authorities, thus it might be difficult to set additional criteria.

## Discussion

The approach to improving priority setting using 'accountability for reasonableness' was accepted by decision makers in all the workshops. These decision makers have been bombarded over the past decade by technically-based tools for priority setting that they felt were insufficient, opaque, did not include relevant people, and did not take into account relevant values. Consequently, they viewed the 'accountability for reasonableness' approach with enthusiastic favour because it was the first framework that directly addressed their concerns. Some district participants were concerned that higher level authorities must be sensitized so that they can facilitate implementation at the district level, but these concerns may be alleviated by the findings from the Dar es Salaam workshop at which high level Ministry participants were also very supportive of the approach.

Since for many this was a new approach to improving priority setting, some misunderstandings appeared. The misconceptions need to be continually addressed until planners and other stakeholders can make optimal use of the approach. It is hoped that they may see it as an extra support to the trend toward decentralization and good governance, occurring almost everywhere.

In addition, sensitization to the 'accountability for reasonableness' approach must go beyond the health sector. It was clear to workshop participants that local council members and other politicians should be brought on board. This will enhance acceptance and prepare the councilors and other political figures regarding how decisions on priorities are made.

Previous research has addressed each of the participants' concerns. First, the specifics of the approach may be new to many, though the underlying concepts (e.g. reason-based, transparency, responsiveness) may be familiar. In the mid 1990s Neurosurgeons at Groote Schuur Hospital in Cape Town, South Africa were challenged by a rising number of severe head injuries and reductions in operating time, ICU nurses and beds. Using a process that was reason-based, multi-stakeholder and transparent, the clinicians initiated a collaborative effort to develop a morally defensible resuscitation policy[13]. It has been shown in developed countries that an ongoing, iterative describe-evaluate-improve approach will help build capacity and increased confidence over time [14,15]. Similar research-based improvement initiatives are underway in Chile, Ghana, South Africa, and Zimbabwe [see [16]]. Kapiriri evaluated priority setting using accountability for reasonableness' in several contexts of the Ugandan health system and identified opportunities for improvement[17,18]. These data will likely yield interesting cross-context comparisons. Second, stakeholder involvement can be problematic, particularly in contexts where genuine democratic deliberation is novel. However, nothing but full stakeholder involvement, including patients and members of the public, will ensure that the full range of relevant values are considered [19]. Lessons from other contexts include: keep groups together long enough to allow capacity building[20]. leadership/chairing is key to creating the environment that enables effective stakeholder participation [21], and addressing power imbalances is an absolute requirement for effective stakeholder participation[22]. Third, reason-developing and reason-giving is very complex. Previous research that framed priority setting as a 'trade-off' between competing values was too simplistic and abstract, and underestimated the complexity of reasoning [e.g. [23]]. Priority setting decisions involve complex clusters of many considerations that are shaped by the specific institution and process in which they work and are often decision-specific [24]. As previously noted, approaches to priority setting in developing countries over the past 15 years have emphasized CEA. Though occasionally helpful, formal cost-effectiveness is seldom an overriding consideration[25]. Moreover, "simple solutions", such as CEA, are theoretically flawed and difficult to implement in practice[26].

How do decision makers know when they 'get it right'? Though there is no overarching moral framework that specifies THE right answer, emphasizing transparency and publicity helps ensure that reasons are exposed for examination and challenge, providing a mechanism for improving the quality of the decisions[27]. Moreover, discriminatory decisions on the part of priority setting decision makers can be redressed through the legal system, which provides protection against discrimination.

## Conclusion

This initiative focused on improving priority setting through capacity building with district planning teams to enhance their implementation of the 'accountability for reasonableness' approach. Participants viewed the 'accountability for reasonableness' approach with enthusiastic favour because it was the first framework that directly addressed their concerns. Research is ongoing to evaluate the improvements gained after the approach was implemented.

## Competing interests

The author(s) declare that they have no competing interests.

## Authors' contributions

All authors participated in conducting the initiative reported here, and contributed to the writing and revising of this manuscript. The paper was conceived by DKM.

## Pre-publication history

The pre-publication history for this paper can be accessed here:


